# Diversity of Rhizosphere Soil Arbuscular Mycorrhizal Fungi in Various Soybean Cultivars under Different Continuous Cropping Regimes

**DOI:** 10.1371/journal.pone.0072898

**Published:** 2013-08-20

**Authors:** Weiguang Jie, Xiaorui Liu, Baiyan Cai

**Affiliations:** 1 College of Life Sciences, Heilongjiang University, Harbin, China; 2 Department of Food and Environment Engineering, Heilongjiang East University, Harbin, China; 3 Key Laboratory of Microbiology, Heilongjiang Province, Harbin, China; Graz University of Technology (TU Graz), Austria

## Abstract

Recent studies have shown that continuous cropping in soybean causes substantial changes to the microbial community in rhizosphere soil. In this study, we investigated the effects of continuous cropping for various time periods on the diversity of rhizosphere soil arbuscular mycorrhizal (AM) fungi in various soybean cultivars at the branching stage. The soybean cultivars Heinong 37 (an intermediate cultivar), Heinong 44 (a high-fat cultivar) and Heinong 48 (a high-protein cultivar) were seeded in a field and continuously cropped for two or three years. We analyzed the diversity of rhizosphere soil AM fungi of these soybean plants at the branching stage using morphological and denaturing gradient gel electrophoresis (DGGE) techniques. The clustering analysis of unweighted pair-group method with arithmetic averages (UPGMA) was then used to investigate the AM fungal community shifts. The results showed that increasing the number of years of continuous cropping can improve the colonization rate of AM fungi in different soybean cultivars at the branching stage. The dominant AM fungi in the experimental fields were *Funneliformismosseae* and *Glomus* spp. The number of years of continuous cropping and the soybean cultivar both had obvious effects on the diversity of AM fungi, which was consistent with the results of colonization rate analysis. This study establishes a basis for screening dominant AM fungi of soybean. In addition, the results of this study may be useful for the development of AM fungal inoculants.

## Introduction

Soybean is the fourth largest grain crop in China. In addition, soybean is an important oil crop with high nutritional value. Soybean is extremely rich in soy proteins, which have greater nutritional value than cereal proteins, and it is therefore an important source of plant protein for human consumption [1–3]. Furthermore, soybean also contains active substances that are beneficial for human health, such as isoflavones, saponins and oligosaccharides [4–6].

A large portion of the soybean crop in China has been grown by continuous cropping for many years. This practice can be harmful, as it makes plants susceptible to pest damage and adversely affects the physicochemical properties of soil [7,8]. This can eventually lead to substantial reductions (70%–80%) or even total crop failure, which can result in huge losses in soybean production [3,9]. Yield reduction and quality degradation caused by continuous cropping of soybean have become a global problem. Several studies have focused on the mechanisms underlying the adverse effects of continuous cropping [10,11]. Recent studies have shown that continuous cropping of soybean causes substantial changes to the microbial community of rhizosphere soil [12,13]. This practice causes gradual transformation of the soil from “bacterial type” high fertility soil to “fungi type” low fertility soil. In addition, continuous cropping turns neutral soil into acidic soil, which enhances fungal growth while inhibiting the proliferation of bacteria and actinomycetes. This process results in the conversion of fungi into the dominant community [14,15]. Moreover, continuous cropping for many years causes the enrichment of root exudates, such as phenolic acids, in the soil [13]. Studies have shown that when the concentration of phenolic acids is artificially increased in soil, the quantity of fungi in the soil increases exponentially [16,17]. In addition, continuous cropping of soybean causes an increase in organic compound (sugars, amino acids, organic acids) content in soil and promotes the growth of root rot pathogens [13]. Most sterilization techniques for killing spores indirectly leads to the dominance of fungal colonies in continuously cropped soils [18,19].

Arbuscular mycorrhizal (AM) fungus is a type of oligotrophic microorganism that can infect the root cells of plants and form a symbiotic relationship with plants to promote the growth plants. AM fungi have a wide infection range but feature host-specific dynamic [20–22]. AM fungi in the rhiosphere soil can contribute to the following processes: (1) significantly improve the absorption and utilization efficiency of inorganic nutrient elements in plants [23]; (2) strengthen the adversity and disease resistance of host plants [24,25]; (3) improve the growth environments for host plants; (4) promote community succession; (5) play an active role in ecosystem stability, thereby functioning as a “biofertilizer” [26,27]. Recent studies have focused on the application of AM fungi to crops [28–30]. The effects of AM fungi on leguminous crops have been confirmed with sterilized pot experiments and by studying plant growth under ecologically damaged conditions [31,32]. Studies have shown that AM fungi can enhance the ability of soybean to absorb nutritional elements while improving both the nitrogen-fixing ability of *Rhizobium* and the colonization structure in the rhizosphere niche, thus increasing yields and economic efficiency of soybean [33]. Nevertheless, there was no study on the diversity of rhizosphere soil AM fungi in various soybean cultivars under different continuous cropping regimes has thus far been reported.

This study was conducted using three typical soybean cultivars with comparatively large planting areas in Heilongjiang Province, China, namely Heinong 37, Heinong 44 and Heinong 48. We analyzed the diversity of rhizosphere soil AM fungi associated with soybean plants (at the branching stage) grown under different continuous cropping schemes using morphological and denaturing gradient gel electrophoresis (DGGE) techniques. Specifically, unweighted pair-group method with arithmetic averages (UPGMA) clustering analysis of the DGGE profiles was performed to evaluate AM fungal community shifts. Our findings provide an experimental and theoretical insights for screening and application of dominant AM fungi present under continuous cropping of soybean.

## Materials and Methods

### Materials

Three soybean cultivars with relatively large planting areas in Heilongjiang Province, China were selected for this study, including Heinong 37 (an intermediate cultivar with an average protein content of 40.17% and an average fat content of 20.00%; designated HN37), Heinong 44 (a high-fat cultivar with an average protein content of 36.06% and an average fat content of 23.01%; designated HN44) and Heinong 48 (a high protein cultivar with an average protein content 45.23% and an average fat content of 19.50%; designated HN48). The total protein was determined by Kjeldahl method. The total fat content in soybean is determined chemically by using the Soxhlet extraction method.

### Experimental design

This study was performed at the Experimental Station of the Research Institute of Sugar, Industry, Harbin Institute of Technology, China from May to October, 2011. Each soybean cultivar was sown in two fields, one with two years of continuous cropping and one with three. They were designated L2HN37, L2HN44, L2HN48, L3HN37, L3HN44 and L3HN48 to indicate the number of years of continuous cropping and the soybean cultivar. The production management techniques employed in the trial fields were the same as those employed in standard production fields to ensure that the results would have practical significance. Soil-determination of organic matter, nitrogen, Potassium, phosphorus by Ignition method, Kjeldahl method, NaOH melt - flame photometry method, alkali fusion - Mo-Sb Anti spectrophotometric method, respectively. The experiments were repeated in triplicate.

### Sampling and treatment

The samples were collected 60 days after planting (at the branching stage). During sampling, soil surface debris were removed, and then a puncher was used to take random samples in the rhizosphere soil to collect root and soil samples. Furthermore, the roots from the same field were mixed evenly, as were the soil samples. The two root mixtures from each soybean cultivar with different durations of continuous cropping were designated RL2 and RL3, while the corresponding soil samples were designated SL2 and SL3. Partial root samples were washed with sterile water and fixed in Formalin – acetic acid – alcohol (FAA) fixing solution for observation under a microscope (Nikon TS100, Japan). Prior to microscopy, the root samples were subjected to alkaline lysis and acid fuchsin staining, while the soil samples were air-dried in the shade naturally and stored at 4 °C.

### Determining the colonization rates of soybean roots

Fifty fibrous root segments were selected randomly from the root samples, stained, mounted on slides and observed under a microscope. AM fungal infection of each root segment was observed, and the colonization rates were calculated. To minimize random error three experiments were carried out in parallel, and average values determined for each set of parallel experiments.

### PCR-DGGE

Root DNA was isolated as described previously [34], and the DNA in soil was isolated using an Soil DNA Kit (Omega, USA) according to the manufacturer’s instructions. The nested-PCR reaction procedures and the reaction system were as described previously [35]. In the first amplification, primers GeoA2 (5′-CCAGTAGTCATATGCTTGTCTC-3′) and Geo11 (5′-ACCTTGTTACGACTTTTACTTCC-3′) were used to amplify the fungal 18S rDNA sequence using the following program: initial denaturation at 94 °C (4 min); 30 cycles of denaturation at 94 °C (1 min), annealing at 54 °C (1 min), and extension at 72 °C (2 min); final extension at 72 °C (7 min). The target DNA fragments were confirmed based on their length of about 1800 bp [36].

Product from the first amplification was diluted 1/100 and used as template in asecond amplification using the AM fungal specific primer AM1 (5′- GTTTCCCGTAAGGCGCCGAA-3′) in combination with the universal eukaryotic primer NS31(5′-TTGGAGGGCAAGTCTGGTGCC-3′) [37,38]. This amplification was performed in a thermal cycler programmed as follows: initial denaturation at 94 °C (2 min); 30 cycles of denaturation at 94°C (45 s), annealing at 65 °C(1 min) and extension at 72 °C (45 s); final extension at 72 °C (7 min). The target DNA fragments were about 550 bp (Figure S1). Product from the second PCR amplification was diluted 1/100 and used as template in a third reaction using the primers NS31-GC (5′- CGCCCGGGGCGCGCCCCGGGCGGGGCGGGGGCACGGGGGTTGGAGGGCAAGTCTGGTGCC -3′) and Glol (5′- GCCTGCTTTAAACACTCTA -3′) [39]. This amplification step was performed in a thermal cycler programmed as follows: initial denaturation at 94°C (2 min); 30 cycles of denaturation at 94 °C (45 s), annealing at 55 °C (1 min) and extension at 72 °C (45 s); final extension at 72 °C (7 min). The target DNA fragments were about 270 bp (Figure S2). All PCR amplification reactions described in this study were conducted in a 50 μL volume of containing 5.0 µL 10 × PCR buffer (with Mg^2+^), 4.0 µL 2.5 mM dNTPs, 1.0 µL primer 1 (10µM), 1.0 µL primer 2 (10µM), 0.2 µL of Taq DNA polymerase, and 2 μL DNA templates. Amplification products were separated by gel electrophoresis on 1.0% (W/V) agarose gel in TAE buffer.

The total of 3.0 μL of the third PCR products of root and soil samples from each soybean cultivar were used for DGGE analysis. The experimental conditions for DGGE were as follows: 8.0% acrylamide (acrylamide: bis acrylamide = 37.5:1), 40% to 65% denaturant (100% denaturant was 40% formamide and 7 mol·L^-1^ urea), electrophoresis temperature 60°C, electrophoresis voltage 130V, electrophoresis time 9 h and silver staining for 15 min.

### Analysis of DGGE results

Ecosystem diversity is often indicated by the abundance, dominance and diversity indices of samples [40]. The abundance and dominance of DGGE bands were compared using Gel-Pro Analyzer 4.5 (Media Cybernetics Company, USA), and the diversity index was calculated with the following formula:

#### Abundance: representing the number of bands in each lane on the DGGE image

Diversity index (*H*): comprehensive value reflecting the richness and evenness of a species, expressed by the Shannon-Weiner index. The larger the *H* is, the higher the diversity of the ecosystem.

$$H=-\sum_{i=1}^{s}\left(P_{i})(\log_{2}P_{i}\right)$$,

where S is the number of bands on the sample DGGE fingerprint image and P_i_ is the dominance of the *i* ^*th*^ band.

### Sequencing and cluster analysis

Selected bands on the DGGE gel were excised and amplified according to the third nested-PCR reaction system and reaction procedure using the recovery solution as template. The PCR products were ligated to the pGEM-T vector (Tiangen, GER) and transformed into *E. coli* DH 5 (AS, CHN) competent cells. The positive clones were screened, reamplified and analyzed once more using DGGE to verify that the DNA segment inserted into the plasmid was a single-band DNA. Positive clones complying with the DGGE detection requirements were selected for sequencing. The sequencing results were submitted to the GenBank database for BLAST comparison to retrieve sequence information from related species. All nucleotide sequences retrieved in this study were deposited in the GenBank database: JX183559 and JX203231-JX203245. Phylogenetic analysis was performed using DNAMAN 7.0 (Lynnon Biosoft, USA) based on neighbor-joining method. Bootstrap resampling analysis for 1000 replicates was performed to estimate the confidence of tree topologies. UPGMA clustering analysis of the DGGE profiles were performed using GelCompar II software (Applied Maths, BVBA, Sint-Martens-Latem, Belgium).

## Results

### Physicochemical parameters of the analysed soils

The major physicochemical properties of the experimental soils were as follows: organic matter 26.13 g·kg^-1^, total N 1.69 g·kg^-1^, total K 25.4 g·kg^-1^, total P 5.5 g·kg^-1^, alkali-hydrolyzable N 133.1 mg·kg^-1^, available P 13.14 mg·kg^-1^, available K 206 mg·kg^-1^ and pH 7.0.

### The effect of continuous cropping duration and soybean cultivar on the colonization rate of AM fungi

**Figure 1:**
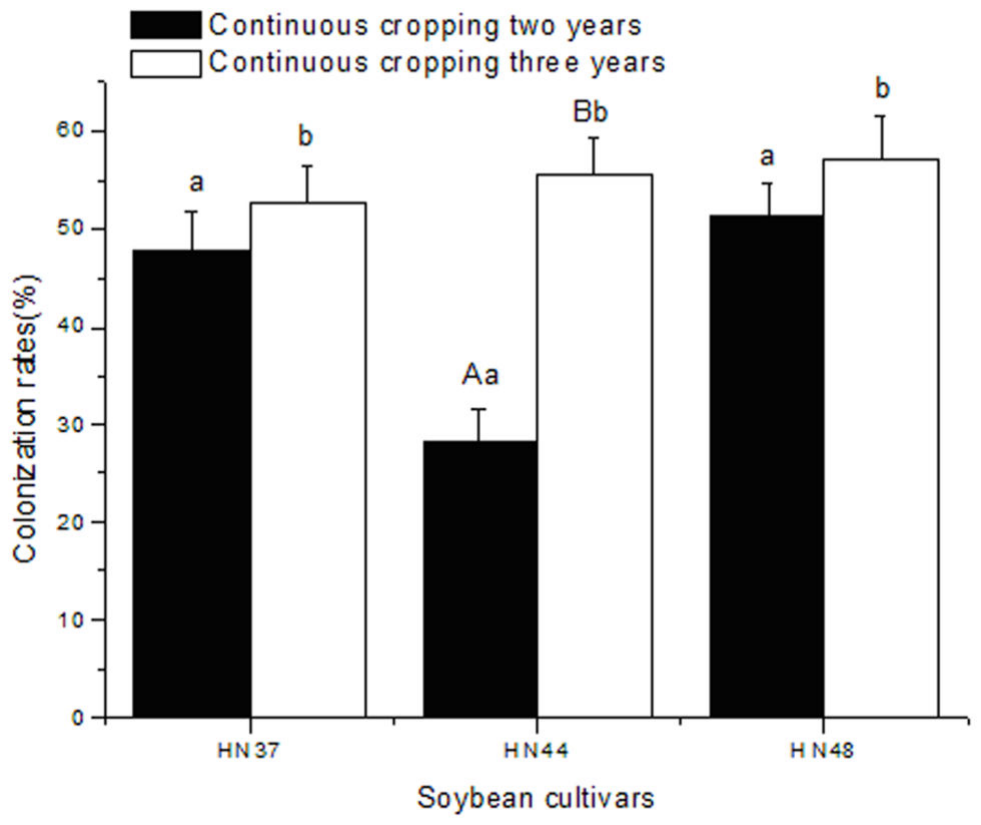
Colonization rate of AM fungi in soybean subjected to different durations of continuous cropping. Error bars represent one standard error. Capital and lower-case letters in the same column indicate the significant difference levels *P* < 0.01 and *P* < 0.05, respectively.

The colonization rates of AM fungi in soybean at the branching stage subjected to different years of continuous cropping were analyzed statistically (Figure 1). According to the data shown in Figure 1, the colonization rate of AM fungi in soybean under three years of continuous cropping at the branching stage was higher than that under two years of continuous cropping, suggesting that increasing the duration of continuous cropping was beneficial for the colonization of AM fungi in soybean roots. This naturally led to an increased colonization rate of AM fungi in the soybean roots.

According to the data shown in Figure 1, when the continuous cropping years of samples were the same, the colonization rates of AM fungi differed depending on the soybean cultivar; HN48 had the highest colonization rate among the cultivars tested. The percent of AM infection in HN37 and HN48 were significantly different (*P* < 0.05), as shown in Figure 1, and that of HN44 differed quite significantly (*P* < 0.01) depending on the years of continuous cropping, according to variance analysis. The three cultivars (*P* < 0.05) were significantly different, according to variance analysis of the cultivars. Therefore, the years of continuous cropping and the soybean cultivar both had substantial effects on the colonization rate of AM fungi in soybean roots. However, the years of continuous cropping had quite significant effects on the colonization rate of AM fungi in soybean roots.

### DGGE analysis of root/soil samples at the branching stage

**Figure 2:**
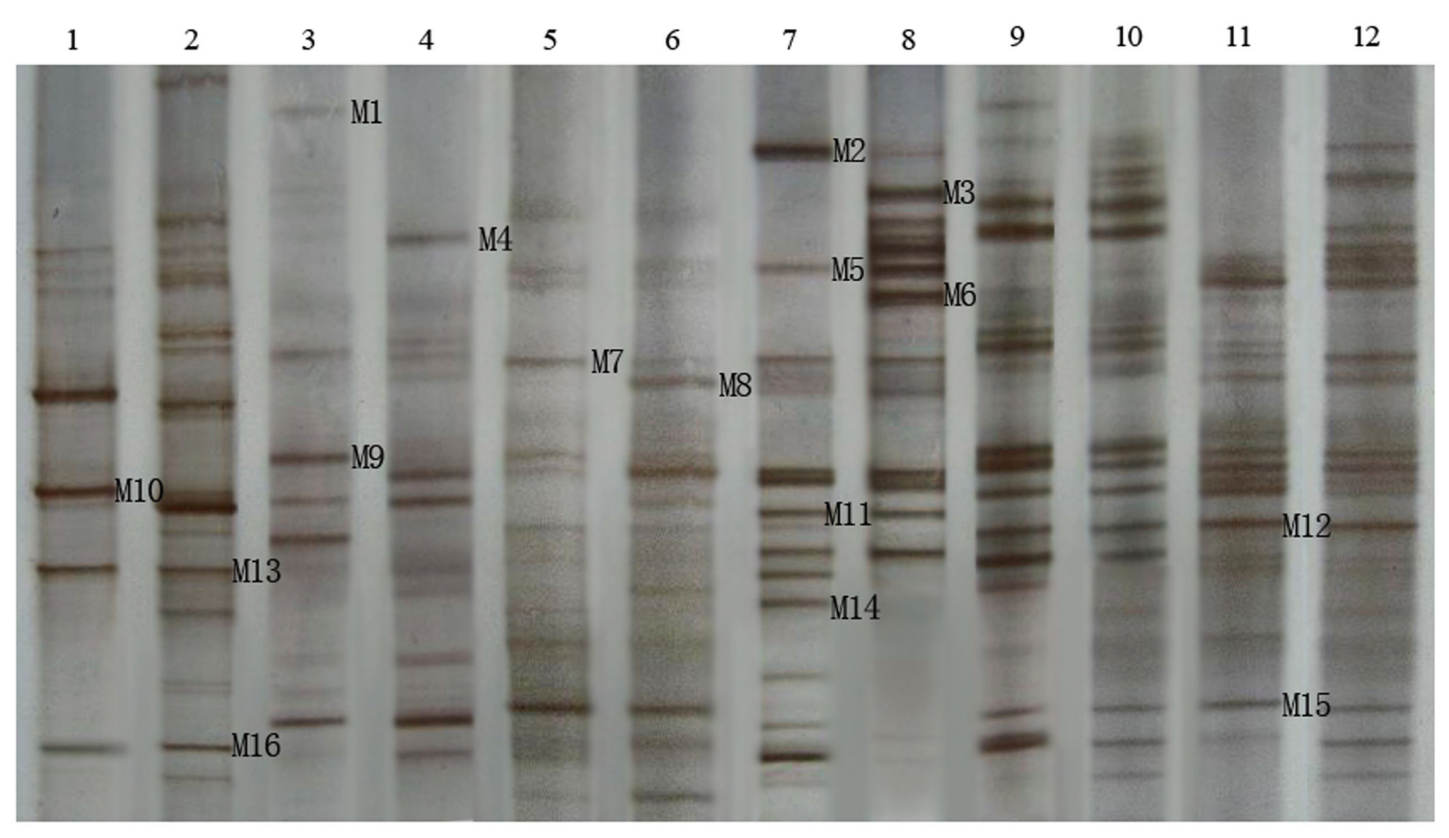
DGGE image of root and soil samples from different soybean cultivars at the branching stage. 1: HN37, root sample, two continuous cropping years; 2: HN37, root sample, three continuous cropping years; 3: HN44, root sample, two continuous cropping years; 4: HN44, root sample, three continuous cropping years; 5: HN48, root sample, two continuous cropping years; 6: HN48, root sample, three continuous cropping years; 7: HN37, soil sample, two continuous cropping years; 8: HN37, soil sample, there continuous cropping years; 9: HN44, soil sample, two continuous cropping years; 10: HN44, soil sample, three continuous cropping years; 11: HN48, soil sample, two continuous cropping years; 12 : HN48, soil sample, three continuous cropping years.

The PCR products of the NS31/Glol region from root and soil samples of three soybean cultivars were subjected to DGGE analysis. The bands were well-separated, exhibiting different DGGE fingerprinting characteristics and brightness (Figure 2), suggesting that the AM fungal community structures were different in all samples. A total of 16 bands (M1-M16) with excellent separation and a high frequency were excised, amplified and subjected to further DGGE analysis to verify that the DNA segment inserted into the plasmid was single-band DNA. The positive clones were then sequenced.

### Phylogenetic analysis

After sequencing, the DNA sequence fragment lengths of all 16 bands were approximately 234 bp. The sequences were submitted to GenBank and compared with homologous sequences. The homologous sequences were all highly similar, with most levels of similarity greater than 97%. The strain represented by the M15 band did not belong to AM fungi, indicating that sequences other than those of AM fungi were also amplified with the NS31/Glo1 primers of 18S rDNA, which suggests that these primers lacked specificity to a certain degree. The community represented by the other 15 bands belonged to AM fungi. The community represented by M2, M8 and M11 shared more than 98% of sequence similarity to *Funneliformismosseae* ; the other 12 bands shared comparatively high similarity with *Glomus* spp., including the community represented by M6 and M12, which shared up to 99% of sequence similarity to *Glomus viscosum* and *Glomus versiforme*. According to the sequencing results, the dominant populations of AM fungi in the rhizosphere soil microbial community structure were mainly presented by *Glomus* and *Funneliformismosseae* .

**Figure 3:**
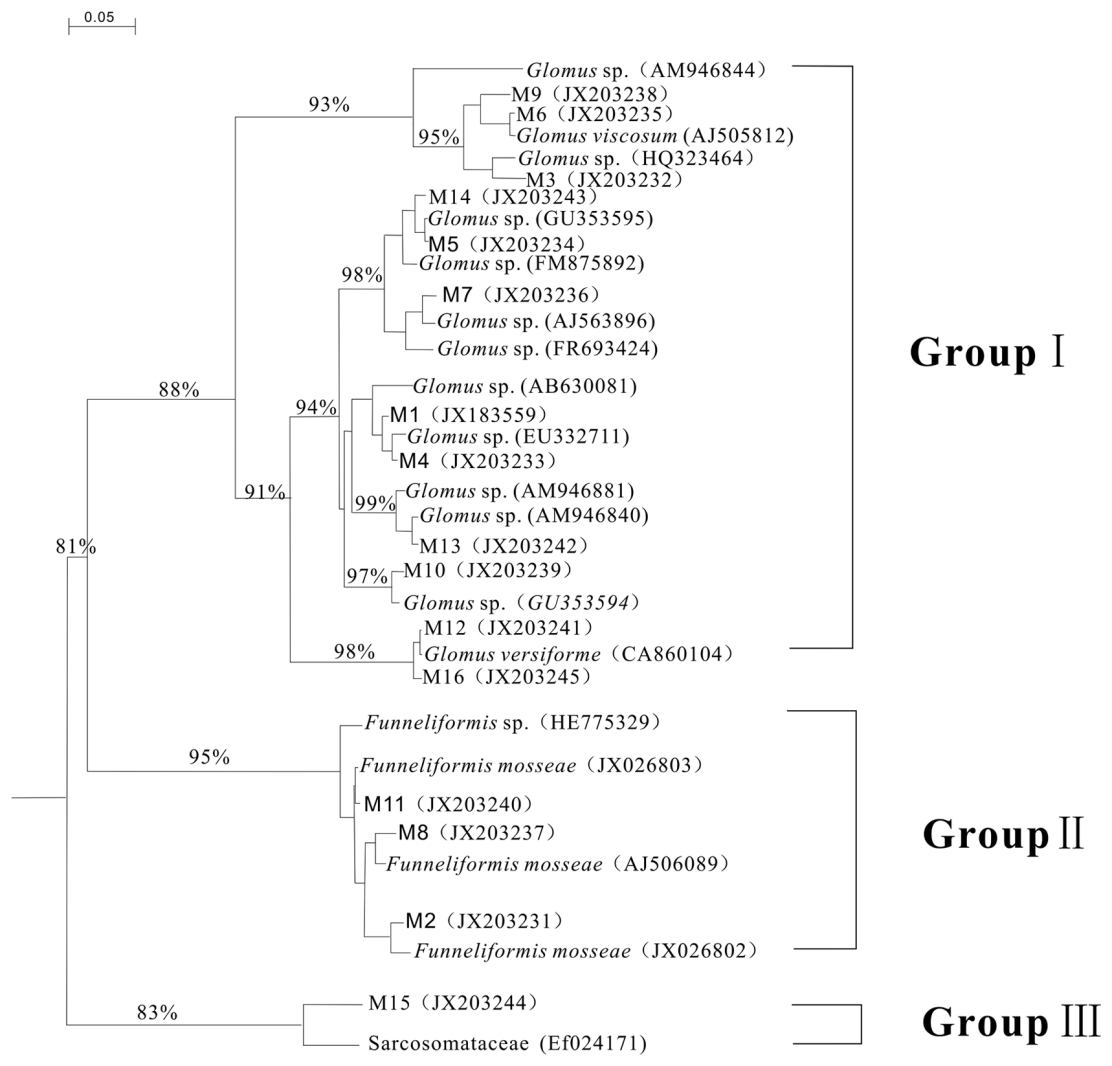
The 16 DGGE sequences and the phylogenetic tree of their closely related species. Phyogenetic tree showing the relatedness of the 18S rDNA NS31/Glo1 domain sequences and other related taxa. Bootstrap values (expressed as percentages of 1000 replications) greater than 80% are shown at branch-points. Bar, 0.05 substitutions per nucleotide position. M1-M16: Sequences obtained from the 16 bands in each lane on the DGGE fingerprints.

A phylogenetic tree was constructed by performing multiple comparisons between the sequences derived from DNAMAN 7.0 and known sequences of most similar strains from the GenBank database (Figure 3).

The data in Figure 3 show that 16 bands from the DGGE image sequencing primarily included three types of community, the first two of which were dominant AM fungi populations in the sample. The first community was *Glomus*, which included the *Glomus* genus and *Glomus versiforme*; the second community was *Funneliformismosseae* and the third community was Sarcosomataceae.

### The effects of continuous cropping on the AM fungi population structure in different soybean cultivars

Statistical examination of the abundance of three soybean cultivars showed that the abundance of each cultivar with three years of continuous cropping was higher than that with two years, indicating that increasing the duration of continuous cropping was beneficial for increasing the abundance of AM fungal community. Variance analysis showed that the abundance of AM fungal community among cultivars subjected to the same duration of continuous cropping significantly differed (Table 1). For example, HN37 and HN48 in SL3 were not significantly different, but both were significantly different from HN44, suggesting that AM fungal community were abundant in HN44 in the rhizosphere soil of soybean at the branching stage in plants subjected to three years of continuous cropping.

**Table 1:** Variance analysis of the effects of different years of continuous cropping on AM fungal community abundance among soybean cultivars.

Sample No.	HN37	HN44	HN48
RL2	6a	9c	8b
RL3	11c	10b	9a
SL2	9a	12c	10b
SL3	12a	13b	12a

Note: Lower-case letters in the same line indicate the significant difference level *p* < 0.05.

Statistical analysis of the diversity indices of three soybean cultivars in both root and soil samples showed that Shannon-Wiener indices were all higher than those of cultivars subjected to two years of continuous cropping, indicating that continuous cropping is beneficial for increasing the diversity index of AM fungal community. Variance analysis showed that the diversity indices of AM fungi community in the rhizosphere soils of different soybean cultivars (at the branching stage) under the same continuous cropping scheme were significantly different (Table 2). HN44 and HN48 in RL2 were not significantly different, but both were significantly different from HN37, suggesting that AM fungi community had a larger diversity index in HN37 roots, and these AM fungi had a more complicated structure under these conditions.

**Table 2:** Variance analysis of the effects of different years of continuous cropping on AM fungal community diversity index among soybean cultivars.

Sample No.	HN37	HN44	HN48
RL2	2.51±0.01a	3.15±0.02bc	3.06±0.02b
RL3	3.53±0.02a	3.53±0.02a	3.55±0.02ab
SL2	3.52±0.03a	3.82±0.03c	3.56±0.03ab
SL3	3.63±0.03a	3.86±0.03c	3.70±0.03ab

Note: Lower-case letters in the same line indicate the significant different level *p* < 0.05.

**Figure 4:**
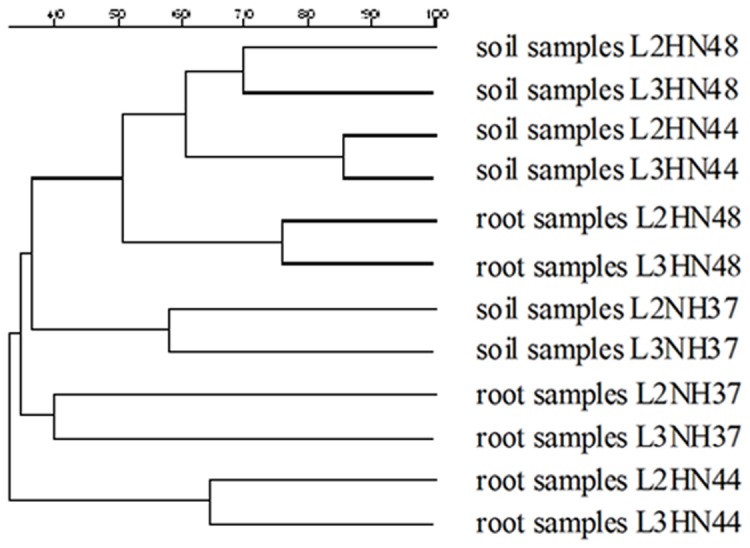
UPGMA cluster analysis of DGGE profiles for root and soil samples from different soybean cultivars at the branching stage.

UPGMA cluster analysis based on DGGE profiles showed that the AM fungal communities were clustered into six groups: Group Ⅰ contained root samples L2NH37 and L3NH37. Group Ⅱ contained root samples L2HN44 and L3HN44. Group Ⅲ contained root samples L2HN48 and L3HN48. Group Ⅳ contained soil samples L2NH37 and L3NH37. Group Ⅴ contained soil samples L2HN44 and L3HN44, which had the lowest similarity. Group Ⅵ contained soil samples L2HN48 and L3HN48 (Figure 4). It revealed that the AM fungal communities structures were significantly affected by soybean cultivars, cropping duration and micro-habitats: soil or roots. The AM fungal communities are cultivar-specific and dependent on the cropping duration.

## Discussion

The failure to produce pure AM fungi cultures is the greatest obstacle for investigation of this system. Recently, the application of PCR-DGGE techniques to microbial ecology studies has led to substantial progress in the study of AM fungi [41]. In the current study, we explored the effects of continuous cropping duration on the colonization rate and microbial community structure of AM fungi in roots and rhizosphere soils of different soybean cultivars at the branching stage using morphological and PCR-DGGE techniques. The results of this study reveal the influence of continuous cropping duration on the AM fungi of soybean cultivars and the dominant AM fungi on different soybean genotypes at the branching stage, providing a theoretical foundation for research examining the relationship between AM fungal inoculants and soil-borne pathogens under continuous cropping conditions, as well as studies leading to new agricultural production guidelines. However, intrinsic problems with the PCR-DGGE technique have limited its application. In this study, the non-AM fungal band M15, which shares up to 91% similarity with that of Sarcosomataceae fungi, was amplified. Furthermore, some studies have shown that the AM primer cannot amplify AM fungi in Archaeosporaceae and Paraglomaceae [42]. Similar finding was obtained in the current study [43,44]. In addition, DGGE fingerprinting approach also has limitations. Shannon-Wiener index calculated for the DGGE profiles underestimates sub-dominant and low-number species.

The physicochemical properties of soils are important factors that affect AM fungal colonization. Continuous cropping of soybean can lead to changes in soil nutrients, soil microorganisms, soil enzyme activities and other factors [45]. Although these changes are not beneficial for the growth of crops such as soybean, resulting in the continuous cropping obstacle, a variety of changes in the rhizosphere soil environment are beneficial for the growth of AM fungi. Continuous cropping can lead to an imbalance in rhizosphere soil nutrients, some of which are in short supply and are essential for soybean growth. This type of environment indirectly increases the colonization rate of AM fungi in soybean roots, thus improving the antagonism effects of AM fungi on the continuous cropping obstacle. Moreover, abiotic parameters and seasonality are important factors that influence AM communities [46]. Schultz reported that AM fungi differ in their seasonality, with some fungi sporulating in late spring and others sporulating at the end of summer [47]. In this study, AM fungi in the soil of a crop grown under different continuous cropping durations exhibited different colonization rates. The colonization rate of AM fungi in HN37 at the branching stage that were subjected to three years of continuous cropping was higher than that grown under two years of continuous cropping, while the diversity index of AM fungi showed similar results, suggesting that continuous cropping of soybean is beneficial for the growth and colonization of AM fungi. This result may be associated with the transition of soil microbial community structure from “bacterial type” to “fungal type” due to the increase in the duration of continuous cropping in such fields. The more barren the soil was, the more efficiently the AM fungi formed mycorrhiza. Furthermore, the results of our study showed that AM fungi community structures in the rhizosphere soils were significantly different among different soybean cultivars at the branching stage. The root-colonized AM fungi were also different, suggesting that AM fungi have different colonization rates among different soybean cultivars. Therefore, it is important to consider differences in the AM fungal community in rhizosphere soils of different soybean cultivars. The function of AM fungi in promoting plant growth must be fully exploited to reduce damage to soybean production caused by continuous cropping.

## Supporting Information

Figure S1 **Second PCR amplification results from root and soil samples of three soybean cultivars.** M: DL2000 Marker; 1–2: HN37 root samples PCR; 3–4: HN44 root samples PCR; 5–6: HN48 root samples PCR; 7–8: HN37 soil samples PCR; 9–10: HN44 soil samples PCR; 11–12: HN48 soil samples PCR.
(TIF)

Figure S2 **Third PCR amplification results of root and soil samples from three soybean cultivars.** M: DL2000 Marker; 1–2: HN37 root samples PCR; 3–4: HN44 root samples PCR; 5–6: HN48 root samples PCR; 7–8: HN37 soil samples PCR; 9–10: HN44 soil samples PCR; 11–12: HN48 soil samples PCR.
(TIF)

## References

[1] Jin J, Liu XB, Wang GH, Mi L, Shen ZB et al. (2010) Agronomic and physiological contributions to the yield improvement of soybean cultivars released from 1950 to 2006 in Northeast China. Field Crops Res 1: 116-123.

[2] Hou J, Zhang PD, Yuan XZ, Zheng YH (2011) Life cycle assessment of biodiesel from soybean, jatropha and microalgae in China conditions. Renew Sust Energ Rev 9: 5081-5091.

[3] Liu XB, Li YS, Han BJ, Zhang QY, Zhou KQ (2012) Yield response of continuous soybean to one-season crop disturbance in a previous continuous soybean field in Northeast China. Field Crops Res 138: 52-56. doi:10.1016/j.fcr.2012.09.012.

[4] Shao SQ, Duncan AM, Yang R, Marcone MF, Rajcan I et al. (2009) Tracking isoflavones: From soybean to soy flour, soy protein isolates to functional soy bread. J Funct Foods 1: 119-127. doi:10.1016/j.jff.2008.09.013.

[5] Tantry MA, Khan IA (2013) Saponins from *Glycine max* Merrill (soybean). Fitoterapia 87: 49-56. doi:10.1016/j.fitote.2013.03.021. PubMed: 23558204. 23558204 10.1016/j.fitote.2013.03.021

[6] Wang QS, Ying TJ, Jahangir MM, Jiang TJ (2012) Study on removal of coloured impurity in soybean oligosaccharides extracted from sweet slurry by adsorption resins. J Food Eng 111: 386-393. doi:10.1016/j.jfoodeng.2012.02.005.

[7] Einhellig FA (1995) Mechanism of action of allelochemicals in allelophthy. Allelopathy J 1: 97-115.

[8] Baziramakenga R (1995) Effects of benronic and cinnamic acids on membrability of soybean roots. Chem Ecol 10: 1272-12810. 10.1007/BF02027561 24234626

[9] Wang SQ, Han XZ, Qiao YF, Wang SY, Li XH (2009) Variation of Soil Enzymes Activity and Relevant Nutrients at Different Years of Soybean (*Glycine max* L.) Rotation, Alternate and Continuous Cropping. Soybean Sci 4: 611-615 (in Chinese)

[10] Yu ZH, Wang GH, Jin J, Liu JD, Liu XB (2011) Soil microbial communities are affected more by land use than seasonal variation in restored grassland and cultivated Mollisols in Northeast China. Eur J Soil Biol 6: 357-363.

[11] Gesch RW, Archer DW (2013) Double-cropping with winter Camelina in the northern Corn Belt to produce fuel and food. Ind Crops Prod 44: 718-725. doi:10.1016/j.indcrop.2012.05.023.

[12] Meriles JM, Gil SV, Conforto C, Figoni G, Lovera E et al. (2009) Soil microbial communities under different soybean cropping systems: Characterization of microbial population dynamics, soil microbial activity, microbial biomass, and fatty acid profiles. Soils Till Res 103: 271-281. doi:10.1016/j.still.2008.10.008.

[13] Guo ZY, Kong CH, Wang JG, Wang YF (2011) Rhizosphere isoflavones (daidzein and genistein) levels and their relation to the microbial community structure of mono-cropped soybean soil in field and controlled conditions. Soil Biol Biochem 11: 2257-2264.

[14] Vargas Gil S, Meriles J, Conforto C, Basanta M, Radl V et al. (2011) Response of soil microbial communities to different management practices in surface soils of a soybean agroecosystem in Argentina. Eur J Soil Biol 1: 55-60.

[15] Tanaka Y, Watanabe J, Mogi Y (2012) Monitoring of the microbial communities involved in the soy sauce manufacturing process by PCR-denaturing gradient gel electrophoresis. Food Microbiol 1: 100-106. PubMed: 22475947. 10.1016/j.fm.2012.02.005 22475947

[16] Ley RE, Schmidt SK (2002) Fungal and bacterial responses to phenolic compounds and amino acids in high altitude barren soils. Soil Biol Biochem 34: 989-995. doi:10.1016/S0038-0717(02)00032-9.

[17] Francis FG, Dalel S, Geoff MM, Roger M (1998) The effect of phenolic acids and molasses spent wash concentration on distillery wastewater remediation by fungi. Proc Biochem 33: 799-803. doi:10.1016/S0032-9592(98)00050-8.

[18] Pedersen P, Lauer JG (2004) Soybean growth and development response to rotation sequence and tillage system. Agron J 96: 1005-1012. doi:10.2134/agronj2004.1005.

[19] Abdallah YEY (2012) Effect of plant traps and sowing dates on population density of major soybean pests. J Basic App Zool 1: 37-46.

[20] Daniel LM, Pedro MA, Matthias CR (2009) Arbuscular mycorrhizal fungi pre-inoculant identity determines community composition in roots. Soil Biol Biochem 6: 1173-1179.

[21] Whiteside MD, Digman MA, Gratton E, Treseder KK (2012) Organic nitrogen uptake by arbuscular mycorrhizal fungi in a boreal forest. Soil Biol Biochem 55: 7-13. doi:10.1016/j.soilbio.2012.06.001. 10.1016/j.soilbio.2012.06.001 PMC3871874 24371363

[22] Eisenhauer N, König S, Alexander CWS (2009) Impacts of earthworms and arbuscular mycorrhizal fungi (Glomus intraradices) on *plant performance are not interrelated* . Soil Biol Biochem 3: 561-567.

[23] Vaz ABM, Sampedro I, Siles JA (2012) Arbuscular mycorrhizal colonization of Sorghum *vulgare* in presence of root endophytic fungi of *Myrtus communis* . Appl Soil Ecol 61: 288-294. doi:10.1016/j.apsoil.2011.10.017.

[24] Asghari HR, Cavagnaro TR (2012) Arbuscular mycorrhizas reduce nitrogen loss via leaching. PLOS ONE 1: e29825 PubMed: 22253790. 10.1371/journal.pone.0029825 PMC3254610 22253790

[25] Whiteside MD, Garcia MO, Treseder KK (2012) Amino acid uptake in arbuscular mycorrhizal plants. PLOS ONE 10: e47643 PubMed: 23094070. 10.1371/journal.pone.0047643 PMC3475604 23094070

[26] Andrés PS, María LSM, Andrés PP, Rosario A (2009) Arbuscular mycorrhizal fungi increased growth, nutrient uptake and tolerance to salinity in olive trees under nursery conditions. J Plant Physiol 13: 1350-1359. 10.1016/j.jplph.2009.02.010 19342122

[27] Feddermann N, Finlay R, Thomas B (2010) Functional diversity in arbuscular mycorrhiza – the role of gene expression, phosphorous nutrition and symbiotic efficiency. Fungal Ecol 1: 1-8.

[28] Tan FX, Wang JW, Chen ZN, Feng YJ, Chi GL et al. (2011) Assessment of the arbuscular mycorrhizal fungal community in roots and rhizosphere soils of Bt corn and their non-Bt isolines. Soil Biol Biochem 12: 2473-2479.

[29] Golubski AJ (2011) Dual plant host effects on two arbuscular mycorrhizal fungi. Pedobiologia 4: 209-216.

[30] Hart MM, Forsythe JA (2012) Using arbuscular mycorrhizal fungi to improve the nutrient quality of crops; nutritional benefits in addition to phosphorus. Sci Hort Amsterdam 148: 206-214. doi:10.1016/j.scienta.2012.09.018.

[31] Meghvansi MK, Prasad K, Harwani D, Mahna SK (2008) Response of soybean cultivars toward inoculation with three arbuscular mycorrhizal fungi and Bradyrhizobium japonicum in the alluvial soil. Eur J Soil Biol 44: 316-323. doi:10.1016/j.ejsobi.2008.03.003.

[32] Miransari M (2011) Hyperaccumulators, arbuscular mycorrhizal fungi and stress of heavy metals. Biotechnol Adv 29: 645-653. doi:10.1016/j.biotechadv.2011.04.006. PubMed: 21557996. 21557996 10.1016/j.biotechadv.2011.04.006

[33] Tian H, Drijber RA, Zhang JL, Li XL (2013) Impact of long-term nitrogen fertilization and rotation with soybean on the diversity and phosphorus metabolism of indigenous arbuscular mycorrhizal fungi within the roots of maize (*Zea mays* L.). Agric Ecosyst Environ 164: 53-61. doi:10.1016/j.agee.2012.09.007.

[34] Long LK, Yang SZ, Yao Q, Zhu HH (2005) DNA extraction from arbuscular mycorrhizal fungi and analysis by PCR-denaturing gradient gel electrophoresis. Mycosystema 4: 564-569 (in Chinese)

[35] Cai BY, Ge JP, Jie WG, Yan XF (2009) The community composition of the arbuscular mycorrhizal fungi in the rhizosphere of *Phellodendron amurense* . Mycosystema 4: 512-520 (in Chinese)

[36] Schwarzott D, Schüβler A (2001) A simple and reliable method for ssuRNA gene DNA extraction, amplification and cloning from single AM fungal spore. Mycorrhiza 10: 203-207. doi:10.1007/PL00009996.

[37] Helgason T, Daniell TJ, Husband R, Fitter AH, Yong JPW (1998) Ploughing the word-wide-web? Nature 384: 431. 10.1038/28764 9697763

[38] Simon L, Lalonde M, Bruns TD (1992) Specific amplification of l8S fungi ribosomal genes from vesicular arbuscular endomycorrhizai fungi colonizing roots. Appl Environ Microbiol 58: 291-295. PubMed: 1339260. 1339260 10.1128/aem.58.1.291-295.1992 PMC195206

[39] Comejo P, Azcón-Aguilar C, Barea JM, Ferrol N (2004) Temporal temperature gradient gel electrophoresis (TTGE) as a tool for the characterization of arbuscular mycorrhizal fungi. FEMS Microbiol Lett 241: 265-270. doi:10.1016/j.femsle.2004.10.030. PubMed: 15598542. 15598542 10.1016/j.femsle.2004.10.030

[40] Lv XC, Weng X, Zhang W, Rao PF, Ni L (2012) Microbial diversity of traditional fermentation starters for Hong Qu glutinous rice wine as determined by PCR-mediated DGGE. Food Control 28: 426-434. doi:10.1016/j.foodcont.2012.05.025.

[41] Liang ZB, Drijber RA, Lee DJ, Dwiekat IM, Harris SD et al. (2008) A DGGE-cloning method to characterize arbuscular mycorrhizal community structure in soil. Soil Biol Biochem 40: 956-966. doi:10.1016/j.soilbio.2007.11.016.

[42] Daniell TJ, Husband R, Fitter AH, Young JPW (2001) Molecular diversity of arbuscular mycorrhizal fungi colonising arable crops. FEMS Microbiol Ecol 36: 203-209. doi:10.1111/j.1574-6941.2001.tb00841.x. PubMed: 11451525. 11451525 10.1111/j.1574-6941.2001.tb00841.x

[43] Ma WK, Siciliano SD, Germida JJ (2005) A PCR-DGGE method for detecting arbuscular mycorrhizal fungi in cultivated soils. Soil Biol Biochem 9: 1589-1597.

[44] Powell JR, Campbell RG, Dunfield KE, Gulden RH, Hart MM et al. (2009) Effect of glyphosate on the tripartite symbiosis formed by Glomus intraradices, Bradyrhizobium japonicum, and genetically modified soybean. Appl Soil Ecol 1: 128-130.

[45] Zhao YW, Bi DM, Zhao QZ, Liu CZ, Hu ZY (2006) Physiological and ecological effects of sulfur fertilization on soybean. Chin J Appl Ecol 12: 2376-2380 (in Chinese) 17330483

[46] Bever JD, Schultz PA, Pringle A, Morton JB (2001) Arbuscular mycorrhizal fungi: more diverse than meets the eye, and the ecological tale of why. BioScience 51: 923-932.

[47] Schultz PA, Bever JD, Morton J (1999) *Acaulospora colossica* sp. nov. from an old field in North Carolina and morphological comparisons with similar species, *A. laevis* and *A. koskei* . Mycologia 91: 676-683. doi:10.2307/3761255.

