# Supplementary figures and images for: Diversity of Rhizosphere Soil Arbuscular Mycorrhizal Fungi in Various Soybean Cultivars under Different Continuous Cropping Regimes
Source: PLoS One. 2013 Aug 20;8(8):e72898. doi: 10.1371/journal.pone.0072898 (PMC3748124; doi:10.1371/journal.pone.0072898)

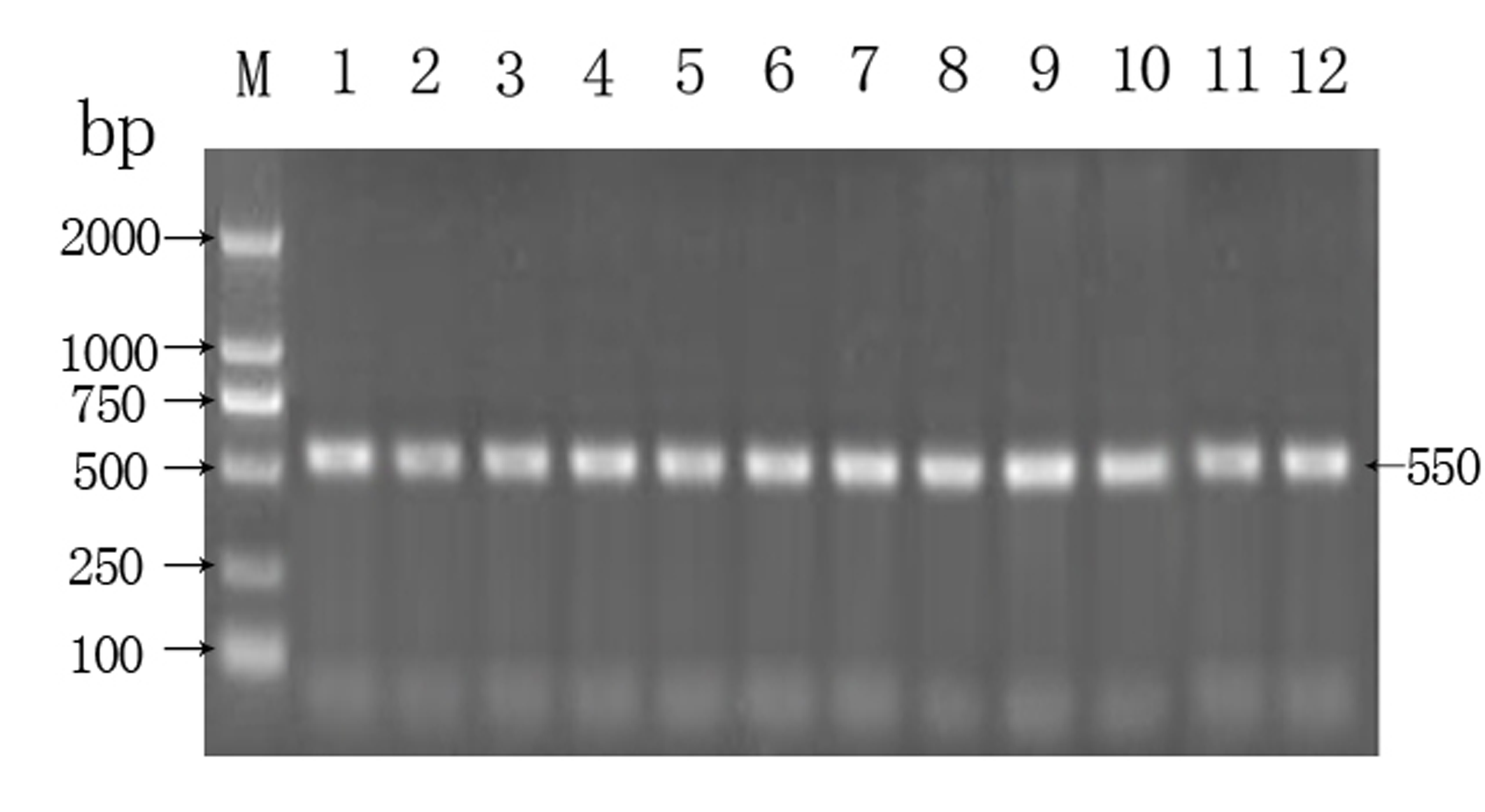

Supplement: Figure S1 — Second PCR amplification results from root and soil samples of three soybean cultivars. M: DL2000 Marker; 1–2: HN37 root samples PCR; 3–4: HN44 root samples PCR; 5–6: HN48 root samples PCR; 7–8: HN37 soil samples PCR; 9–10: HN44 soil samples PCR; 11–12: HN48 soil samples PCR. (TIF) [file pone.0072898.s001.tif]

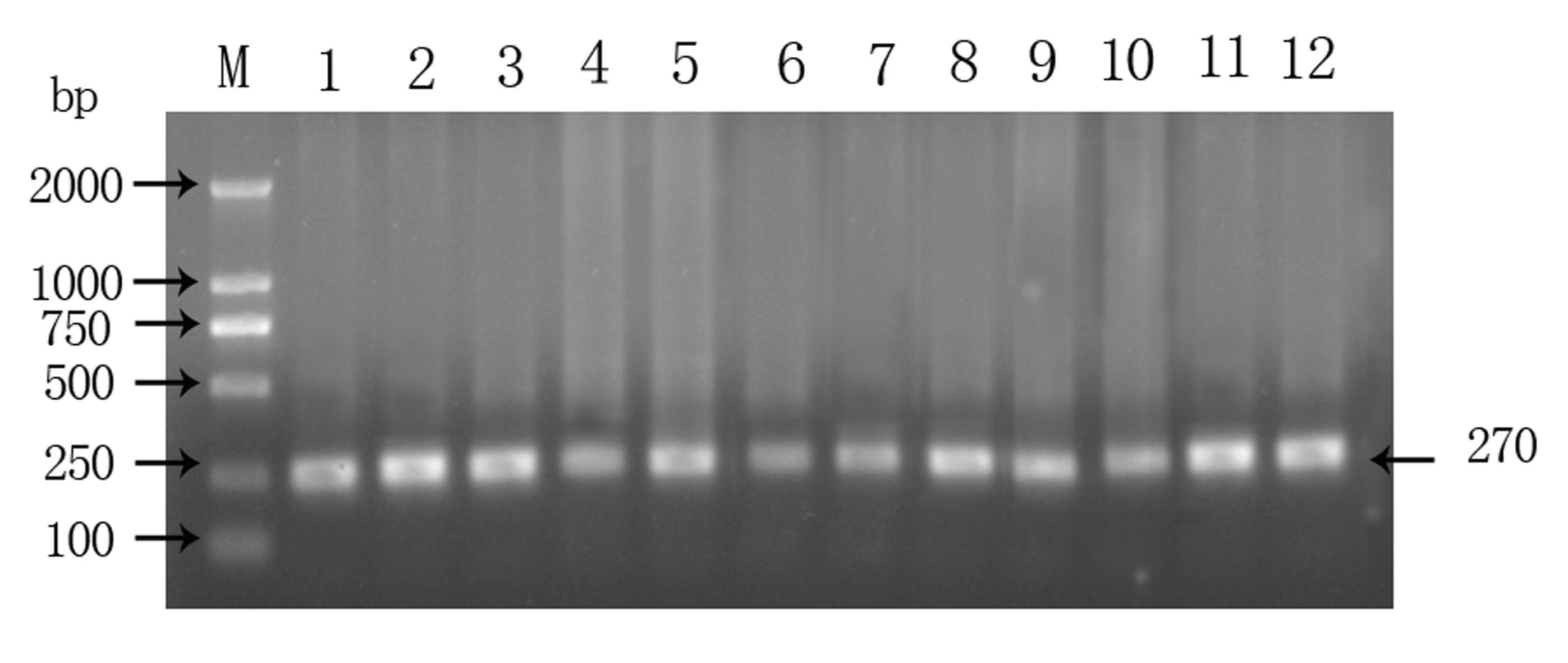

Supplement: Figure S2 — Third PCR amplification results of root and soil samples from three soybean cultivars. M: DL2000 Marker; 1–2: HN37 root samples PCR; 3–4: HN44 root samples PCR; 5–6: HN48 root samples PCR; 7–8: HN37 soil samples PCR; 9–10: HN44 soil samples PCR; 11–12: HN48 soil samples PCR. (TIF) [file pone.0072898.s002.tif]
